# Biochemical markers of endothelial injury in children with obesity—from adhesion molecules to novel endothelium-specific biomarkers

**DOI:** 10.3389/fped.2026.1862008

**Published:** 2026-07-22

**Authors:** K. Madej-Świątkowska, D. Drozdz

**Affiliations:** 1 Doctoral School of Medical and Health Sciences, Jagiellonian University Medical College, Cracow, Poland; 2 Department of Pediatric Nephrology and Hypertension, Jagiellonian University Medical College, Cracow, Poland; 3 Department of Pediatric Nephrology and Hypertension, University Children's Hospital, Cracow, Poland

**Keywords:** biomarkers, cardiovascular risk, childhood obesity, endothelial dysfunction, pediatrics

## Abstract

**Introduction:**

Childhood obesity is no longer only a metabolic condition but an early vascular disease. Subclinical endothelial dysfunction develops long before overt cardiovascular complications and may contribute to the later emergence of atherosclerosis and hypertension. Detecting these early alterations remains a major challenge in pediatric care.

**Methods:**

This mini review integrates current evidence on circulating biochemical markers of endothelial injury in children with obesity, focusing on markers reflecting endothelial activation, hemostatic imbalance, and vascular dysfunction.

**Results:**

Multiple biomarkers are altered in pediatric obesity. Soluble adhesion molecules reflect early endothelial activation and low-grade inflammation. Hemostatic markers indicate a shift toward a prothrombotic state. Dysregulation of the NO–eNOS–ADMA axis contributes to reduced nitric oxide bioavailability and impaired vascular function. Emerging endothelium-specific biomarkers, including endocan and syndecan-1, offer greater specificity and appear to capture early vascular injury more sensitively than traditional inflammatory markers. Indirect markers further link metabolic, inflammatory, and oxidative pathways.

**Conclusions:**

Endothelial dysfunction represents an early, clinically silent but potentially reversible stage of obesity-related vascular disease in children. A multimarker approach may improve early risk identification and better reflect the complexity of underlying mechanisms. These findings support a shift toward earlier, multidisciplinary cardiovascular risk assessment in pediatric obesity and highlight the need for prospective studies to translate biomarkers into clinical practice.

## 1 Introduction

Childhood obesity has become a serious global public health issue. The proportion of children and adolescents aged 5–19 affected by overweight and obesity increased significantly, from 8% in 1990 to 20% in 2022, which translates to more than 390 million individuals worldwide (1). Body mass index (BMI) remains a widely used screening tool for obesity, and obesity is traditionally defined as a BMI exceeding the age- and sex-adjusted norms by more than 2 standard deviations (2, 3). However, recent recommendations emphasize that BMI should not be used as a stand-alone measure of obesity at the individual level; confirmation of excess adiposity requires additional anthropometric assessment or direct body fat measurement, followed by clinical evaluation when appropriate (3). Excess body weight in early life increases the risk of metabolic disorders, including hypertension, dyslipidemia, and insulin resistance, and promotes the premature development of atherosclerosis, leading to a higher cardiovascular risk in adulthood (4, 5). The atherosclerotic process may begin in childhood and is associated with chronic inflammation and endothelial dysfunction related to oxidative stress and pro-inflammatory cytokine overproduction (2, 4). Given the rarity of overt cardiovascular events in the pediatric population, identifying subclinical markers of endothelial dysfunction is essential for early risk stratification and the timely implementation of preventive strategies (6). The aim of this review is to summarize current evidence on biochemical markers of endothelial injury in children with obesity and to evaluate their potential clinical relevance.

Previous reviews have discussed endothelial dysfunction in obesity mainly in adult populations, whereas pediatric data remain relatively limited and heterogeneous. In children and adolescents, less attention has been given to endothelial-specific and glycocalyx-related biomarkers and their possible relevance to early vascular alterations. Therefore, this mini-review provides an updated overview of endothelial dysfunction biomarkers in pediatric obesity, with emphasis on their mechanistic background and potential clinical implications.

## 2 Pathophysiology

The vascular endothelium, despite its single-layer structure, is a highly specialized metabolic and endocrine organ that regulates vascular homeostasis by controlling vascular tone, blood flow, hemostasis, and inflammatory responses (7, 8). Its functional integrity depends on paracrine and autocrine signaling, as well as on the endothelial glycocalyx, which maintains vascular barrier function and limits leukocyte–endothelium interactions (7).

Endothelial dysfunction is strongly linked to oxidative stress. When reactive oxygen and nitrogen species (ROS and RNS) are produced in excess, the level of nitric oxide (NO) decreases, despite the fact that it is the primary vasodilator synthesized by endothelial nitric oxide synthase (eNOS) (7, 9). NO is essential for maintaining vascular homeostasis, as it modulates vascular tone and prevents platelet aggregation and leukocyte adhesion (9).

Under conditions of increased oxidative stress, eNOS uncoupling occurs, whereby the enzyme produces superoxide anion (O_2_•^−^) instead of NO, leading to the formation of peroxynitrite (ONOO^−^) and enhanced oxidative damage (9). Reduced NO bioavailability disrupts the balance between vasodilatory and vasoconstrictive mediators, impairing regulation of vascular tone (7, 8).

This process involves the activation of inflammatory pathways, including nuclear factor kappa B (NF-*κ*B) and mitogen-activated protein kinase (MAPK). At the same time, adhesion molecules such as VCAM-1 and ICAM-1 are upregulated, promoting leukocyte attachment to the endothelium and enhancing vascular inflammation (8, 9). Prolonged oxidative stress may further promote prothrombotic processes, including increased expression of von Willebrand factor (vWF) (8).

**Figure 1:**
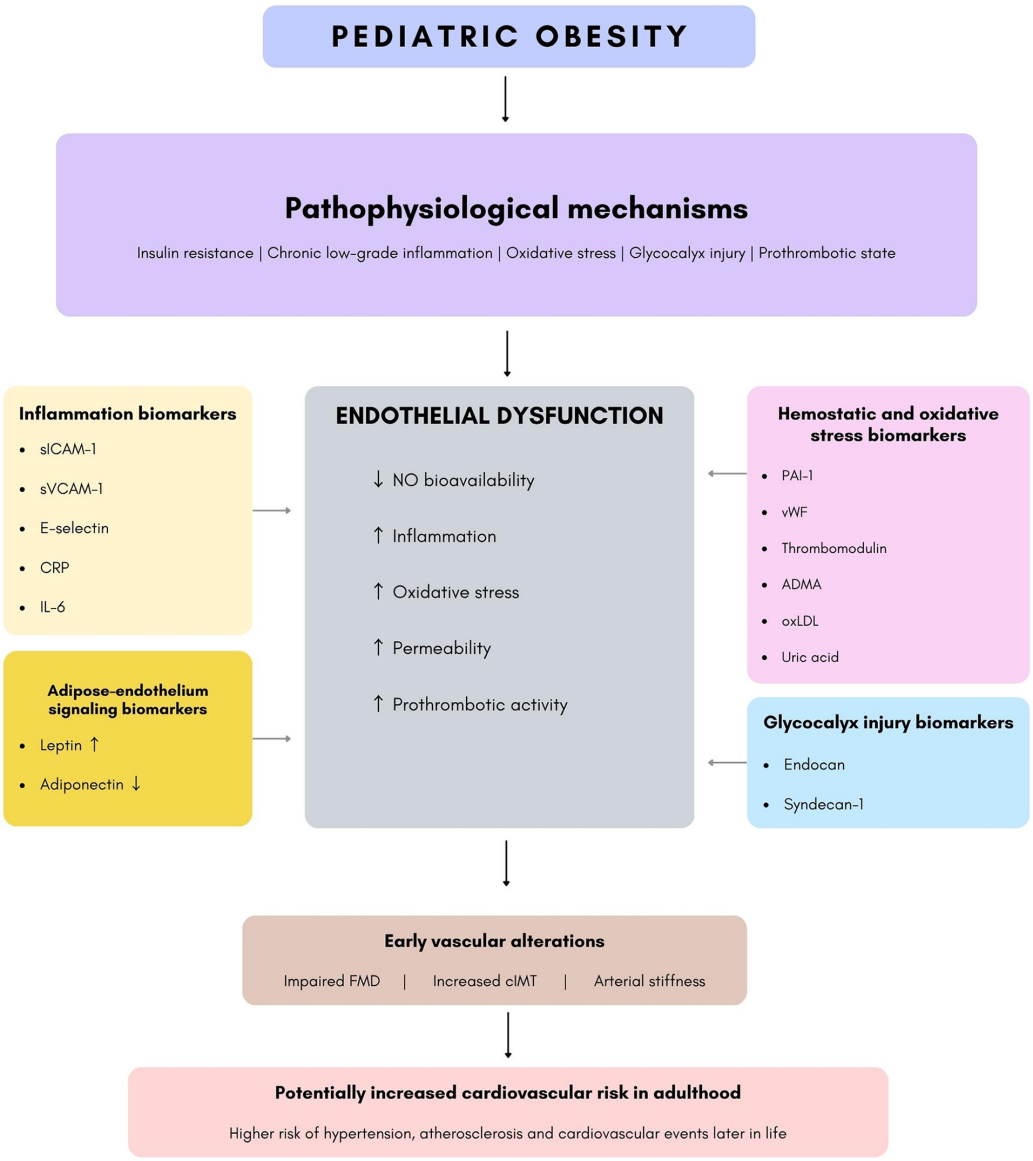
Key biomarkers and mechanisms involved in vascular dysfunction.

As a result, the endothelium shifts from a protective (“vasoprotective”) phenotype to a pro-oxidative, pro-inflammatory, and prothrombotic state, representing mechanisms associated with cardiovascular diseases such as hypertension and atherosclerosis (7–9). Figure 1 summarizes the main mechanisms and biomarkers involved in endothelial dysfunction in pediatric obesity.

## 3 Biochemical markers of endothelial injury

### 3.1 Soluble adhesion molecules (sICAM-1, sVCAM-1, E-selectin)

Soluble adhesion molecules (sICAM-1, sVCAM-1, E-selectin) are among the best-characterized markers of early endothelial activation and injury. Their expression increases in response to inflammatory, metabolic, and hemodynamic stimuli, leading to the release of soluble forms into the circulation (10, 11). These molecules play a key role in leukocyte adhesion and transendothelial migration, and are considered involved in early atherogenesis.

Pediatric studies have consistently demonstrated elevated levels of these markers in children with obesity, hypertension, type 1 diabetes, and chronic kidney disease (6, 10–12). Their concentrations correlate with major cardiovascular risk factors, including BMI, blood pressure, lipid profile, insulin resistance, and inflammatory markers, reflecting the low-grade inflammation characteristic of pediatric obesity. Notably, increased levels have also been observed in children without overt metabolic disturbances, supporting their role as markers of subclinical endothelial dysfunction (10, 12).

sICAM-1 reflects both endothelial activation and sustained injury, showing strong associations with metabolic and inflammatory parameters as well as blood pressure variability (6). sVCAM-1, which is more selective for monocytes and T lymphocytes, is particularly associated with lipid abnormalities, especially low-density lipoprotein (LDL) levels (6, 10). In contrast, E-selectin, a marker of early endothelial activation, is strongly linked to both metabolic and hemodynamic parameters, including mean arterial pressure and arterial stiffness indices (6, 13).

Together, these markers may reflect a characteristic profile of endothelial activation in pediatric obesity and may serve as useful tools for the early detection of vascular dysfunction.

### 3.2 Markers of endothelial hemostatic dysfunction: PAI-1, thrombomodulin, and von willebrand factor (vWF)

Hemostatic disturbances represent a key component of endothelial dysfunction in obesity, shifting the balance between fibrinolysis and coagulation toward a prothrombotic state. As a result, the endothelium loses its antithrombotic properties and adopts a procoagulant phenotype. In pediatric studies, three principal markers have been identified: plasminogen activator inhibitor-1 (PAI-1), soluble thrombomodulin (sTM), and von Willebrand factor (vWF), reflecting different stages of endothelial dysfunction (5, 6, 11).

PAI-1, a major inhibitor of fibrinolysis, is increased in obese children and those with metabolic syndrome, with elevated levels observed already at early stages of obesity (14). Its concentration correlates with metabolic parameters, including body mass index (BMI), waist circumference, lipid profile, fasting glucose, insulin resistance, and blood pressure (14), supporting its association with a metabolically driven prothrombotic endothelial phenotype.

Elevated levels of soluble thrombomodulin (sTM), a marker of endothelial injury, have been observed in obese children, especially when multiple cardiovascular risk factors are present (5, 6, 11). Its levels are associated with adverse hemodynamic and inflammatory profiles, as well as markers of vascular damage such as flow-mediated dilation (FMD) and intima–media thickness (IMT) (11), and with visceral obesity and leptin levels (5).

In contrast, increased vWF levels are observed mainly in children with full metabolic syndrome rather than in those with obesity alone (11, 14), suggesting that elevated vWF may be associated with a more advanced stage of endothelial dysfunction associated with cumulative metabolic disturbances.

Overall, these markers provide complementary information on the extent of endothelial injury, with PAI-1 and sTM appearing more useful for detecting early alterations in the pediatric population.

### 3.3 The NO–eNOS–ADMA axis: a molecular mechanism of endothelial dysfunction

Nitric oxide (NO) is a key endothelium-derived mediator of vascular homeostasis and one of the principal vasodilatory factors produced in the vascular wall. As a gaseous signaling molecule with high diffusibility, it readily crosses cell membranes and acts on vascular smooth muscle cells. Its vasodilatory effect is primarily mediated by activation of soluble guanylyl cyclase, increased cyclic guanosine monophosphate (cGMP) production, and a subsequent reduction in intracellular calcium levels. In addition, NO exerts vasoprotective effects by inhibiting platelet aggregation and adhesion, leukocyte adhesion, and smooth muscle cell proliferation. Reduced NO bioavailability is considered a central feature of endothelial dysfunction and may contribute to increased vascular resistance, vasoconstriction, hypertension, and atherosclerosis (7, 9, 15).

The NO–endothelial nitric oxide synthase (eNOS)–asymmetric dimethylarginine (ADMA) axis plays a crucial role in regulating endothelial function. eNOS catalyzes the synthesis of NO from L-arginine, while ADMA inhibits its activity, leading to decreased NO production. Elevated ADMA levels are associated with impaired endothelium-dependent vasodilation and increased cardiovascular risk, and may occur already in early stages of obesity, preceding overt metabolic and vascular abnormalities (16).

Furthermore, oxidative stress may lead to eNOS uncoupling, in which the enzyme produces reactive oxygen species instead of NO, further impairing endothelial function and accelerating vascular damage (16). These disturbances may be reflected in reduced flow-mediated dilation (FMD), a functional marker of endothelial dysfunction observed in obesity (16).

### 3.4 Novel endothelium-specific biomarkers: endocan (ESM-1) and syndecan-1 (SDC-1)

Endocan (ESM-1) is a soluble proteoglycan secreted by activated endothelial cells of the microvasculature, conferring high endothelial specificity compared to classical inflammatory markers (17). Its expression is upregulated by pro-inflammatory and angiogenic stimuli, as well as metabolic stress and hypoxia, and it is involved in the regulation of leukocyte adhesion, endothelial permeability, and angiogenesis (18).

Available studies consistently demonstrate elevated endocan levels in obese children, particularly in those with metabolic syndrome (2, 17, 18). Endocan is associated with metabolic parameters such as body mass index (BMI), homeostasis model assessment of insulin resistance (HOMA-IR), blood pressure, triglycerides, and glucose levels, and may serve as an independent indicator of insulin resistance (17, 18). Moreover, it has been identified as an independent predictor of increased carotid intima–media thickness (cIMT), linking biochemical and imaging markers of vascular injury (2).

As an endothelium-specific marker, endocan reflects endothelial activation and injury rather than systemic inflammation alone, supporting its potential utility as a biomarker of early vascular dysfunction, particularly when combined with imaging techniques.

Syndecan-1 (SDC-1) is a transmembrane proteoglycan of the endothelial glycocalyx responsible for maintaining vascular barrier integrity and limiting leukocyte and platelet adhesion (19–21). Under inflammatory conditions, glycocalyx degradation and increased shedding of SDC-1 occur, and its circulating form serves as a marker of endothelial injury (2, 4).

In children with obesity, elevated SDC-1 levels are associated with metabolic disturbances and renal function, while visceral adiposity is linked to enhanced glycocalyx degradation, suggesting a potential association with early endothelial glycocalyx injury (2, 4, 19–21).

### 3.5 Indirect markers: uric acid, CRP, IL-6, adipokines, oxLDL

Indirect markers reflect key pathogenic mechanisms underlying endothelial dysfunction in obesity, including insulin resistance, oxidative stress, and chronic low-grade inflammation. Although not endothelium-specific, they are strongly associated with endothelial activation and early atherogenesis (12, 16, 22–24).

Serum uric acid (SUA) is one of the most well-established indirect markers. In obese children, SUA levels are increased and correlate with body mass index (BMI), insulin resistance, triglycerides, and inversely with high-density lipoprotein (HDL) levels (12). In multivariate analyses, SUA remains an independent predictor of inflammatory and endothelial markers such as C-reactive protein (CRP), interleukin-6 (IL-6), and soluble intercellular adhesion molecule-1 (sICAM-1), suggesting a potential association with oxidative stress and reduced nitric oxide (NO) bioavailability (12, 22, 23).

C-reactive protein (CRP) reflects chronic low-grade inflammation and is elevated in pediatric obesity, correlating with metabolic disturbances and hyperuricemia (12). Interleukin-6 (IL-6), although not consistently associated with obesity itself, is associated with insulin resistance and an inflammatory burden, identifying a more adverse metabolic phenotype (12, 24).

Adipokines, including leptin and adiponectin, link adipose tissue dysfunction with endothelial injury. In obesity, leptin may promote oxidative stress and reduce NO bioavailability, whereas decreased adiponectin impairs endothelial nitric oxide synthase (eNOS) activation and the vasoprotective phenotype (16, 17, 23).

Elevated levels of oxidized low-density lipoprotein (oxLDL) are also observed in obese children, independent of low-density lipoprotein (LDL) concentration, indicating enhanced lipoprotein oxidation (25). OxLDL has been implicated in foam cell formation, induces cytotoxic effects in endothelial cells, and contributes to inflammation and vascular remodeling (26, 27). Additionally, obesity-related oxidative stress reduces NO bioavailability, further impairing endothelial function (27).

Overall, these markers integrate metabolic, inflammatory, and oxidative pathways and complement endothelium-specific biomarkers in cardiovascular risk assessment in pediatric obesity.

Key endothelial dysfunction biomarkers discussed in pediatric obesity are summarized in Table 1.

**Table 1:** Biomarkers of endothelial dysfunction in pediatric obesity.

Endothelial process	Biomarker	Main findings in pediatric obesity	Clinical relevance	References
Endothelial activation and inflammation	Soluble intercellular adhesion molecule-1 (sICAM-1)	– associated with adiposity, insulin resistance, systolic blood pressure, and low-grade inflammatory burden – reflects activation of endothelial adhesion pathways and enhanced leukocyte–endothelium interaction	– marker of early inflammatory endothelial activation and cardiometabolic risk – most informative together with blood pressure, insulin resistance, lipid profile, and inflammatory markers	(10, 13, 15)
	Soluble vascular cell adhesion molecule-1 (sVCAM-1)	– linked with dyslipidemia, especially total and LDL cholesterol abnormalities – reflects endothelial activation and monocyte adhesion to activated endothelium, an early step in atherogenesis	– may indicate obesity-related vascular inflammatory activation coupled to dyslipidemia – useful as part of a biomarker panel rather than as a single diagnostic marker – isolated elevation does not distinguish obesity from other inflammatory or vascular conditions	(6, 10, 15)
	E-selectin	– a more endothelial-specific adhesion molecule – observed together with triglycerides, blood pressure, and metabolic/hemodynamic abnormalities – linked in pediatric studies with ambulatory blood pressure load and arterial stiffness parameters, supporting endothelial activation under vascular stress	– potential indicator of early endothelial activation and obesity-related vascular stress – may be particularly informative with hypertriglyceridemia, elevated blood pressure, or impaired glucose metabolism	(10, 13, 15, 29)
Systemic low-grade inflammation affecting the endothelium	C-reactive protein (CRP) Interleukin-6 (IL-6)	– linked with insulin resistance, adiposity, and chronic low-grade inflammatory burden – may amplify oxidative stress, impaired nitric oxide signaling, and endothelial adhesion molecule expression; not endothelial-specific	– widely accessible markers of obesity-related inflammatory burden and cardiometabolic risk – do not directly measure endothelial injury without corroborating endothelial or vascular markers	(12, 15, 24)
Impaired fibrinolysis and prothrombotic shift	Plasminogen activator inhibitor-1 (PAI-1)	– correlated with visceral adiposity, BMI, waist circumference, insulin resistance, hypertriglyceridemia, glucose levels, and blood pressure – reflects impaired fibrinolysis and a metabolically driven prothrombotic phenotype	– may help identify children with increased prothrombotic and cardiometabolic risk – most meaningful when combined with insulin resistance, dyslipidemia, vWF, platelet activation, or endothelial injury markers	(14, 30)
Loss of anticoagulant endothelial surface properties	Soluble thrombomodulin	– reflects endothelial surface injury, shedding of anticoagulant membrane components, and loss of local vasoprotective properties – associations with blood pressure, ADMA, oxidized LDL, renal dysfunction, left ventricular mass index have been reported	– suggests more advanced endothelial surface injury rather than simple activation – potentially relevant in obesity with hypertension, renal involvement, or clustered cardiometabolic risk – pediatric obesity-specific data remain limited for routine clinical use	(5, 11, 15)
Endothelial/platelet activation and hemostasis	von Willebrand factor (vWF)	– more consistently increased in metabolic syndrome than uncomplicated obesity – correlated with platelet activation markers such as sP-selectin – links reported with hyperleptinemia, higher uric acid, and lower HDL cholesterol, supporting endothelial–platelet activation	– potential marker of thrombo-inflammatory vascular risk and hemostatic disturbance – may indicate enhanced platelet–endothelium interaction, especially in metabolic syndrome or clustered cardiometabolic risk – more suggestive of advanced vascular/hemostatic abnormality than isolated early endothelial activation	(11, 14, 30)
Impaired vasodilation and reduced nitric oxide bioavailability	NO–eNOS–ADMA axis	– obesity-related insulin resistance and oxidative stress may reduce NO bioavailability through altered eNOS signaling and increased ADMA activity – linked with impaired flow-mediated dilation and endothelial dysfunction in pediatric obesity – – NO–endothelin-1 imbalance linked with elevated blood pressure and early vascular dysregulation in adolescents	– reflects impaired vasodilatory reserve and altered vascular homeostasis – best interpreted with functional vascular assessment, such as FMD or reactive hyperemia, and ADMA/NO metabolites where available	(7, 9, 15, 16, 31, 32)
Oxidative lipid modification and early atherogenic injury	Oxidized LDL (oxLDL)	– accompanied by adiposity, insulin resistance, and oxidative stress; in some studies independent of LDL cholesterol concentration – promotes endothelial injury, macrophage recruitment, and foam-cell formation, linking dyslipidemia with early atherogenesis	– provides information beyond the conventional lipid profile by capturing oxidative lipoprotein modification – potentially useful for high-risk phenotyping, especially in severe obesity, insulin resistance, or metabolic syndrome – currently used mainly in research settings	(25–27)
Metabolic endothelial stress and oxidative-inflammatory signaling	Serum uric acid	– linked with BMI, triglycerides, insulin resistance, blood pressure, CRP, IL-6, and sICAM-1 – independently associated with platelet activation together with leptin, vWF, and HDL cholesterol in obese children	– readily available indirect marker of a metabolic–vascular phenotype with higher thrombo-inflammatory risk – elevated uric acid strengthens concern when clustered with insulin resistance, dyslipidemia, or elevated blood pressure – not proof of endothelial injury and should not be presented as a direct endothelial biomarker	(12, 30)
Endothelial glycocalyx disruption and endothelial-specific activation	Endocan (ESM-1)	– endothelial-derived proteoglycan involved in inflammation, endothelial permeability, cell migration, and adhesion – reported to be elevated in pediatric metabolic syndrome; related to BMI, HOMA-IR, systolic blood pressure, triglycerides, glucose levels, and lower HDL cholesterol – associations with carotid intima–media thickness reported, supporting links with early vascular alterations	– promising endothelial-specific biomarker for obesity-related vascular and metabolic dysfunction – may help identify obesity-related vascular dysfunction associated with insulin resistance and elevated blood pressure – pediatric reference ranges and thresholds for clinical decision-making remain insufficiently established	(2, 6, 17, 18)
	Syndecan-1 (SDC-1)	– glycocalyx component released during endothelial surface layer degradation – elevated SDC-1 levels correlated with metabolic disturbances, triglycerides, and markers of glycocalyx degradation in pediatric obesity – in adolescents with excess weight, correlated positively with creatinine and urea and inversely with estimated glomerular filtration rate, suggesting early renal-endothelial involvement	– potential marker of endothelial glycocalyx injury and microvascular dysfunction – relevant when discussing subclinical renal-endothelial involvement, dyslipidemia, or metabolic syndrome in adolescents with excess weight – clinical role remains investigational; further pediatric validation required	(2, 4, 19)
Adipose tissue–endothelium and platelet crosstalk	Leptin/adiponectin	– related to adiposity, insulin resistance, triglycerides, uric acid, lower HDL cholesterol, and endothelial dysfunction – leptin independently associated with platelet activation; adiponectin shows an opposite, vasoprotective and insulin-sensitizing profile	– helps define the metabolic–vascular phenotype of pediatric obesity – high leptin/low adiponectin pattern supports an insulin-resistant, pro-inflammatory, and prothrombotic risk profile – adipokines should be interpreted as upstream modulators rather than direct markers of endothelial injury	(5, 16, 17, 30)

ADMA, asymmetric dimethylarginine; BMI, body mass index; cIMT, carotid intima–media thickness; CRP, C-reactive protein; eNOS, endothelial nitric oxide synthase; FMD, flow-mediated dilation; HDL, high-density lipoprotein; HOMA-IR, homeostasis model assessment of insulin resistance; IL-6, interleukin-6; LDL, low-density lipoprotein; NO, nitric oxide; oxLDL, oxidized low-density lipoprotein; SDC-1, syndecan-1; vWF, von Willebrand factor.

### 3.6 Clinical translation, limitations, and future perspectives

From a translational perspective, biochemical biomarkers of endothelial dysfunction should currently be viewed mainly as research and risk-stratification tools rather than routine diagnostic tests in pediatric obesity. Most available evidence comes from cross-sectional pediatric cohorts that associate endothelial biomarkers with obesity-related metabolic disturbances such as insulin resistance, dyslipidemia, hypertension, visceral adiposity, and metabolic syndrome. Higher concentrations of adhesion molecules, PAI-1, soluble thrombomodulin, ADMA, endocan, syndecan-1, inflammatory markers, uric acid, and oxLDL have also been observed in these settings. Some of these biomarkers have additionally been linked with vascular imaging parameters, including impaired flow-mediated dilation (FMD), increased carotid intima–media thickness (cIMT), and arterial stiffness indices, suggesting relationships between circulating biomarkers and early vascular dysfunction or remodeling. Taken together, current evidence indicates that endothelial biomarkers may reflect subclinical vascular alterations before clinically overt vascular disease becomes apparent. At the same time, their direct prognostic value for adult cardiovascular outcomes in pediatric populations remains unclear, largely because long-term prospective studies are still limited (2, 11, 15, 17, 28).

Many currently available studies involve relatively small and heterogeneous pediatric cohorts, which makes direct comparison between studies difficult and reduces generalizability of the findings. Interpretation of the evidence is further complicated by differences in biomarker selection, laboratory methods, and study design. Their broader clinical use is also limited by incomplete assay standardization, variable availability, cost considerations, and the lack of validated age-, sex-, and puberty-specific reference ranges or diagnostic cut-off values (8). In addition, some biomarkers show limited endothelial specificity and may be influenced by systemic inflammation or metabolic disturbances independent of vascular injury. For this reason, combining biochemical biomarkers with clinical assessment and vascular imaging may currently be the most practical approach for cardiovascular risk stratification in children with obesity. Further studies are needed to determine whether these biomarkers improve long-term cardiovascular risk prediction and whether they respond to lifestyle or pharmacological interventions.

## 4 Conclusion

Current data suggest that childhood obesity is linked to the early onset of subclinical endothelial dysfunction, a process that plays a central role in the development of atherosclerosis and hypertension and may occur before overt cardiovascular disease. This phenomenon is multifactorial and involves endothelial inflammation, disturbances in hemostasis, and reduced vasoprotective capacity.

Among the reviewed biomarkers, soluble adhesion molecules (sICAM-1, sVCAM-1, sE-selectin) consistently demonstrate elevated levels in obese children and correlate with the severity of metabolic disturbances, making them useful indicators of early endothelial activation. Markers of endothelial hemostasis, particularly plasminogen activator inhibitor-1 (PAI-1) and soluble thrombomodulin, provide additional insight into the extent of endothelial injury, whereas disturbances in the nitric oxide–endothelial nitric oxide synthase–asymmetric dimethylarginine (NO–eNOS–ADMA) axis reflect key molecular mechanisms underlying vascular dysfunction. Endocan, due to its high endothelial specificity, is a particularly promising biomarker, potentially offering greater sensitivity for detecting early vascular alterations than traditional inflammatory markers.

Importantly, this review integrates classical, hemostatic, and emerging endothelium-specific biomarkers, highlighting their complementary roles in assessing endothelial dysfunction in pediatric obesity.

However, none of the currently available biomarkers is sufficiently specific or sensitive to serve as a standalone diagnostic tool. Important limitations include heterogeneity of study populations, methodological variability, and lack of assay standardization, which hinder direct comparison across studies. Moreover, most available data are cross-sectional, limiting causal inference.

From a clinical perspective, a panel-based approach integrating markers of endothelial activation, injury, and metabolic-inflammatory background appears most justified and may improve identification of children at high cardiovascular risk. Future research should focus on prospective validation of biomarkers, assessment of their prognostic value, and integration with imaging modalities such as carotid intima–media thickness (cIMT) and pulse wave velocity (PWV). Early identification of endothelial dysfunction may support earlier risk stratification and targeted preventive interventions in this population.
